# Effects of Mental Illness Amongst Adults in the United States Living With Diabetes Mellitus on Hospital Admissions

**DOI:** 10.7759/cureus.46145

**Published:** 2023-09-28

**Authors:** Marina Gettas, Jim E Banta, R. Patti Herring, W. Lawrence Beeson, Jisoo Oh, Razaz Shaheen

**Affiliations:** 1 Health Policy and Leadership Program, School of Public Health, Loma Linda University, Loma Linda, USA; 2 Health Promotion and Education Program, School of Public Health, Loma Linda University, Loma Linda, USA; 3 Epidemiology and Biostatistics Programs, School of Public Health, Loma Linda University, Loma Linda, USA; 4 Epidemiology and Health Policy and Leadership Programs, School of Public Health, Loma Linda University, Loma Linda, USA; 5 Preventive Care Program, School of Public Health, Loma Linda University, Loma Linda, USA

**Keywords:** diabetes mellitus, united states, adult, chronic disease, comorbidity, hospitalization, psychological distress

## Abstract

Objective: To examine the influence of comorbid mental illness on hospitalization among adults reporting diabetes mellitus.

Methods: This cross-sectional observational study used National Health Interview Survey (NHIS) data from 2000-2018 to examine hospitalization. Mental illness was defined as no to low psychological distress (NLPD), moderate psychological distress (MPD), and serious psychological distress (SPD) as per the Kessler-6 scale. Socio-demographic factors and health status were added as covariates in binary logistic regression.

Results: This study involved 48,807 survey participants and reflected an estimated population of 17,524,418 adults with diabetes in the United States, of whom 19.9% were hospitalized in the year prior to the survey. Among those who were hospitalized, 71.5% exhibited None to Low Psychological Distress (NLPD), 17.7% reported Moderate Psychological Distress (MPD), and 10.8% reported Serious Psychological Distress (SPD). Conversely, among non-hospitalized individuals, the percentages were as follows: 83.2% had NLPD, 11.4% had MPD, and 5.3% had SPD. The odds ratio (OR) for hospitalization was found to be OR=1.31 (95% CI: 1.20, 1.43, p<0.0001) for MPD and OR=1.42 (95% CI: 1.28, 1.58, p<0.0001) for SPD, in comparison to those with no or low psychological distress.

Conclusion: Among adults with diabetes mellitus, those with mental illness were more likely to be hospitalized than those without mental illness. Programs and policies to improve care among adults with both mental illness and diabetes may help to reduce hospitalizations.

## Introduction

The United States (U.S.) spends more on health care than any other nation, yet has an excessive number of individuals who cannot afford health insurance [1]. Consequently, vulnerable populations with chronic diseases such as diabetes mellitus, mental illness, hypertension, and cancer who cannot afford medical care often are not motivated to maintain their health until their problems become severe and require expensive care in a hospital setting [2]. In the U.S., roughly 60% of adults suffer from at least one chronic disease, and about 40% live with at least two chronic diseases (comorbidities) [3]. Management of chronic diseases is the leading cause of the estimated 75% of the $2 trillion spent on chronic illness [2]. Diabetes mellitus and mental illness in particular represent a large proportion of the healthcare cost burden [3].

The projected expenditure of diabetes mellitus in the U.S., type 1 (T1) and type 2 (T2) combined, would roughly be $237 billion every year on direct medical costs and another $90 billion including healthcare costs and loss of productivity costs [4]. Serious mental illness costs about $193.2 billion in lost revenue in the U.S. per year [4]. It was predicted that by 2030, mental illness in the U.S. will cost roughly $6 trillion in healthcare costs and missed wages [5].

In 2016, there were roughly 30 million adults diagnosed with diabetes mellitus. It was estimated that 95% of diagnosed diabetes mellitus cases were type 2 diabetes mellitus (T2DM), and 5% of adults were diagnosed with type 1 diabetes mellitus (T1DM ) [6]. In 2018, about 20% of individuals reported having a mental disorder at some point during the year [7]. Severe mental illness is a long-term mental illness that affects the accomplishment of daily activities and overall good life outcomes [7].

Factors required for proper disease management in such patients include following through with a primary care physician on an outpatient basis, coordinating services to address social and financial obstacles, and health education about proper medication use. Failure to adequately address any of these factors can precipitate hospitalization [8]. Healthcare organizations are suffering a resource burden as more people are identified and treated for comorbidities like diabetes and mental illness [8]. Not only does the complexity of one disease lead to higher healthcare costs, but cost also increases as individuals suffer from more than one disease (i.e., comorbidities) [9]. As treatment and management become more complex, the potential for hospitalization increases, and it is estimated that hospitalization can cost an average of $10,000 for a two-night stay [10]. As the number of individuals who suffer from mental illness increases, so do hospitalizations [11]. Similar to mental illness, it was reported in 2015 that nearly 19% of hospitalizations were due to a complication of diabetes mellitus, and of these, over 10% were readmitted for hospitalization within 30 days [12].

Unfortunately, the number of individuals suffering from both diabetes mellitus and mental illness combined is likely underestimated due to a lack of doctor visits and blood work to indicate diabetes mellitus and under-reporting of mental health symptoms due to potential cultural stigma.

Understanding the hospitalization usage amongst this growing comorbid population will help existing programs and policies to identify obstacles to prevention, care, and maintenance for this comorbid population. In this study we investigated the influence of comorbid mental illnesses, as measured by psychological distress on hospitalization among adults diagnosed with diabetes mellitus.

## Materials and methods

This cross-sectional, observational study used secondary data from the National Health Interview Survey (NHIS), 2000-2018. The study was approved by the Loma Linda University Institutional Review Board (IRB# 5190080). NHIS is used to provide health information on the U.S. civilian non-institutional population through confidential household interviews [13]. This data collection was conducted by the Centers for Disease Control and Prevention (CDC). Within each state in the U.S., a random sampling was performed. The research design employed stratified random sampling, as determined by the CDC. The NHIS dataset included sampling weights, ensuring that the results obtained were representative of the entire nation. NHIS data cover a broad range of health topics and survey results have been used to monitor health status and access to healthcare across the U.S. over time [13]. The sampling design includes an area sample frame for each housing unit based on the most recent U.S. Census report, state stratification, and an oversampling of Black, Hispanic, and Asian Americans [14]. Subjects were reached primarily through the mail and often randomly selected by telephone number and invited to participate in the hour-long household survey [13], with a response rate averaging 70% [15]. 

NHIS data were modified to eliminate all personal information and identifiers before being released for unrestricted use [15]. NHIS data for this study were obtained through the Integrated Public Use Microdata Series (IPUMS) Health Surveys [16]. Multiple years of data were used to obtain a sufficient sample size since a relatively small percentage of the U.S. population have both diabetes and serious mental illness.

**Figure 1:**
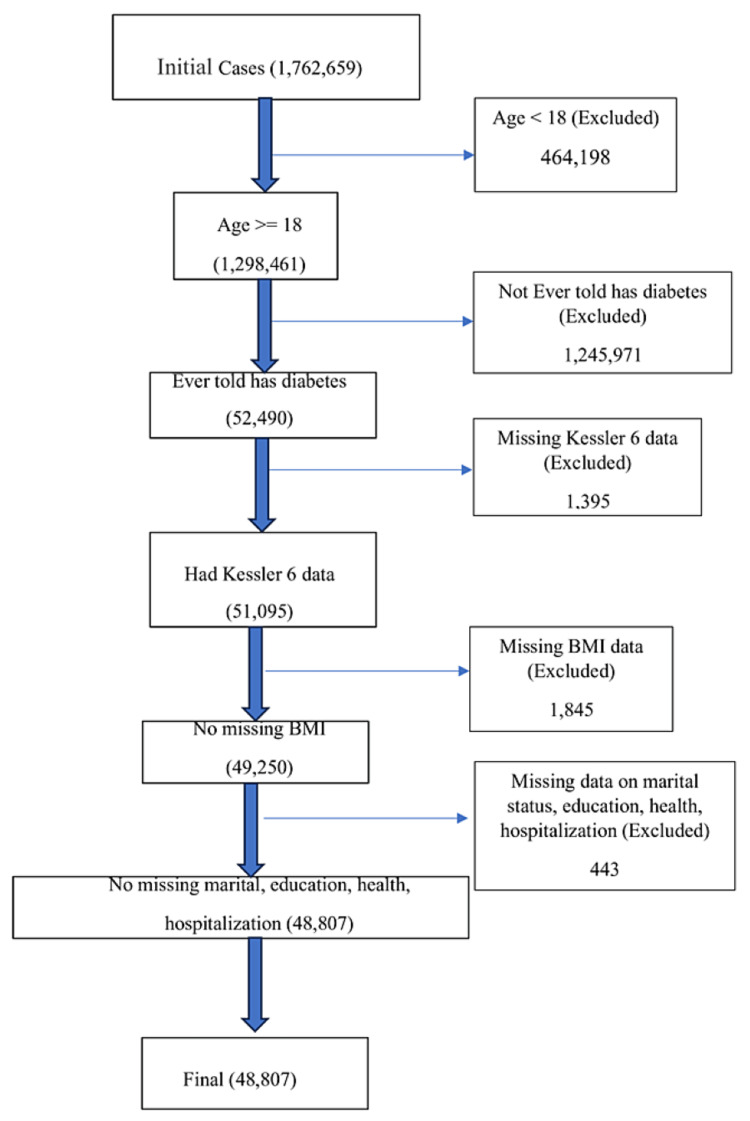
Information about the included and excluded participants.

Study subjects were adults (ages 18 and older) who reported having been diagnosed with “diabetes or sugar diabetes” by a doctor or other health professional. Moreover, only participants who responded to the mental health screening questions were included in this study. Participants must have been able to understand and provide informed consent. Figure 1 provides information about the included and excluded participants.

The Kessler-6 scale was the key measure of mental health status [17]. The National Center for Health Statistics (NCHS) developed and supported the creation of the Kessler-10 scale which was subsequently redesigned by the NHIS to form the Kessler-6 scale [17]. This scale was used to define serious psychological distress. The Kessler-6 scale was originally validated by a convenience sample study in Boston and was found to be as effective as the Kessler-10 scale [18]. Questions include: 1) how often individuals feel nervous; 2) how often individuals feel hopeless; 3) how often individuals feel restless/fidgety; 4) how often individuals feel depressed to the point where nothing can cheer them up; 5) how often individuals feel as if everything is an effort; and 6) how often the individual felt worthless in the past 30 days. Answers ranged from 0 to 4 with the total score ranging between 0 and 24. A score of 13 or higher is used to define serious psychological distress. An additional category of mild or moderate psychological distress, with a score of 5 to 12, has also been validated. A score of 0 to 4 is determined to represent no or low psychological distress [19].

Variables of interest

All the variables, except for age and body mass index (BMI), were already categorical. Age and BMI were converted to categorical variables due to small frequencies within some categories. Education and income categories were also collapsed to present fewer or broader categories for data reduction and efficient presentation in tabular format. Health-related measures included BMI levels, smoking status, and taking insulin shots (yes/no) as a measure of diabetes severity. The outcome was hospitalizations in the prior year (yes/no).

Statistical analysis

We selected variables from our literature review to be forced into the model; therefore, we did not use forward or backward stepwise regression. The statistically significant level for the study was set at alpha = .05. Bivariate distributions for binary variables and the chi-squared tests for categorical variables were used to examine the characteristics of adults with diabetes based on mental health status. Multivariable binary logistic regression was used to determine the independent effect of mental illness on hospitalization. All analyses required survey-weighted techniques using the software SAS 9.4 (SAS Institute, Cary, NC, USA). 

## Results

Table 1 presents characteristics of adults (18+) reporting diabetes mellitus between 2000 and 2018. There was an estimated annual population of 17.5 million adults with diabetes, of which 19.9% were hospitalized in the year prior to the survey. Among those who reported hospitalization, 10.8% (95% CI: 9.9, 11.6) were diagnosed with serious psychological distress, approximately twice as much as those who were not hospitalized and had serious psychological distress, at 5.5% (95% CI: 5.0, 5.7). Those 75 years of age and greater accounted for a greater percentage of those who were hospitalized, 21.5% vs. 15.1% not hospitalized for 75 years and older. Furthermore, a greater percentage of those hospitalized had Medicaid and were hospitalized 20.6% vs. 12.5% who had Medicaid but were not hospitalized, compared to those who were hospitalized and had Medicare coverage 57.1% vs. 42.9% who had Medicare but were not hospitalized. On the other hand, Hispanics accounted for a smaller percentage of those who were hospitalized, 11.5% vs. 15.1% non-Hispanic respectively. Therefore, those who were married were more likely to be hospitalized compared to unmarried, 52.3% vs. 59.7%, respectively. There were modest differences by gender and race. Not surprisingly, those with self-reported poor health accounted for more of those who were hospitalized, 24.1% vs. compared to those who were not hospitalized 9.1%, as did those taking insulin and were hospitalized with 39.8% vs. compared to those who were not hospitalized with 25.9%. Data are not shown for totals; but the percentage of adults with diabetes increased steadily over time, with 3.3% of the sample being surveyed in 2000 compared to 7.0% of the sample in 2018.

**Table 1:** Characteristics of adults (18+) reporting diabetes mellitus in the National Health Interview Survey, 2000-2018 (n=48,807, estimated annual N=17,524,418) P <0.05 statistically significant Northeast: ME, NY, NJ, VT, MA, RI, CT, NW, and PA. North Central/Midwest: IL, IN, IA, KS, MI, MN, MO, NE, ND, OH, SD, and WI. South: DE, FL, GA, MD, NC, SC, VA, WV, AL, KY, MI, TN, AR, LA, OK, and TX. West: AK, AZ, CA, HI, ID, MT, NV, NM, OR, UT, WA, and WY. GED: General Education Diploma

	No Hospitalization	95% Confidence Intervals	Hospitalization	95% Confidence Intervals	P-value
	n=(38,608), N=14,037,511 per year (80.1%)		n=(10,199), N=3,486,907 per year (19.9%)		
	%		%		
Mental Illness					<0.0001
None to low psychological distress	83.20	(82.77, 83.76)	71.52	(70.28, 72.75)	
Moderate psychological distress	11.38	(10.97, 11.79)	17.69	(16.69, 18.68)	
Serious psychological distress	5.35	(5.04, 5.64)	10.78	(9.98, 11.59)	
Region					<0.0001
Northeast	16.14	(15.53, 16.74)	17.08	(15.94, 18.22)	
North Central/Midwest	22.80	(22.09, 23.52)	25.44	(24.27, 26.60)	
South	40.43	(39.55, 41.29)	40.78	(39.42, 42.12)	
West	20.62	(19.82, 21.42)	16.70	(15.75, 17.64)	
Sex					<0.0001
Male	51.70	(51.04, 52.35)	48.84	(47.58, 50.08)	
Female	48.29	(47.64, 48.95)	51.16	(49.91, 52.41)	
Age					<0.0001
18-34	5.15	(4.83, 5.47)	4.75	(4.18, 5.32)	
35-44	9.65	(9.23, 10.06)	7.49	(6.79, 8.19)	
45-54	19.47	(18.90, 20.03)	16.79	(15.79, 17.78)	
55-64	27.50	(26.91, 28.10)	24.94	(23.86, 26.02)	
65-74	23.02	(22.50, 23.54)	24.48	(23.49, 25.46)	
75 and older	15.19	(14.74, 15.64)	21.52	(20.53, 22.52)	
Year					0.11
2000	3.15	(2.96, 3.34)	4.09	(3.64, 4.55)	
2001	3.44	(3.23, 3.64)	4.12	(3.67, 4.57)	
2002	3.59	(3.37, 3.81)	3.90	(3.46, 4.33)	
2003	3.66	(3.44, 3.89)	4.54	(4.02, 5.06)	
2004	4.13	(3.89, 4.37)	4.31	(3.86, 4.76)	
2005	4.48	(4.24, 4.71)	4.57	(4.10, 5.04)	
2006	4.73	(4.44, 5.03)	4.68	(4.11, 5.26)	
2007	4.83	(4.51, 5.15)	4.79	(4.19, 5.40)	
2008	5.23	(4.86, 5.59)	5.01	(4.41, 5.61)	
2009	5.69	(5.35, 6.03)	6.17	(5.45, 6.89)	
2010	5.93	(5.61, 6.25)	5.94	(5.34, 6.53)	
2011	5.83	(5.55, 6.11)	6.05	(5.48, 6.62)	
2012	6.00	(5.72, 6.28)	5.86	(5.31, 6.40)	
2013	6.25	(5.98, 6.53)	5.91	(5.30, 6.53)	
2014	6.32	(6.01, 6.63)	5.11	(4.56, 5.65)	
2015	6.45	(6.11, 6.78)	6.35	(5.70, 6.98)	
2016	6.55	(6.14, 6.95)	5.89	(5.25, 6.51)	
2017	6.52	(6.08,6.95)	6.00	(5.32, 6.68)	
2018	7.14	(6.73, 7.56)	6.62	(5.95, 7.30)	
Race					<0.0001
White	76.61	(75.91, 77.31)	76.4	(75.37, 77.44)	
Black/African American	15.35	(14.78, 15.92)	17.28	(16.35,18.21)	
Asian	4.54	(4.24, 4.83)	2.90	(2.48, 3.32)	
Other race	3.45	(3.09, 3.87)	3.40	(2.92, 3.88)	
Hispanic					<0.0001
No	84.89	(84.24, 85.53)	88.53	(87.67, 89.40)	
Yes	15.10	(14.46, 15.75)	11.46	(10.59, 12.32)	
Marital Status					<0.0001
Married, spouse in household unknown	59.66	(58.96, 60.35)	52.31	(51.02, 53.60)	
Separated	2.69	(2.51, 2.87)	3.15	(2.78, 3.52)	
Divorced	11.75	(11.38, 12.12)	14.46	(13.66, 15.26)	
Widowed	12.28	(11.89, 12.67)	16.3	(15.51, 17.09)	
Living with partner	3.92	(3.65, 4.18)	4.17	(3.65, 4.62)	
Never married	9.68	(9.288, 10.07)	9.62	(8.91, 10.34)	
Education					<0.0019
Grade 9-12, no diploma or GED	22.27	(21.67, 22.85)	25.93	(24.84, 27.02)	
High school graduate	30.78	(30.12, 31.42)	30.46	(29.31, 31.59)	
Some college, no 4-year degree	27.62	(27.04 ,28.18)	27.56	(26.43, 28.68)	
Bachelor's degree	11.87	(11.44, 12.29)	10.00	(9.25, 10.76)	
Master's, Professional, or Doctoral Degree	7.47	(7.12, 7.81)	6.03	(5.44, 6.63)	
Federal Poverty Level					<0.0001
At or above poverty threshold	87.89	(87.33, 88.25)	82.87	(81.99, 83.76)	
Below poverty threshold	12.21	11.76, 12.67)	17.13	16.24, 18.01)	
Family’s Home					<0.0001
Owned or being bought	72.69	(72.02, 73.36)	66.18	(64.94, 67.422)	
Rented	24.94	(24.29, 25.60)	30.56	29.31, 31.80)	
Other arrangement	2.37	(2.19, 2.54)	3.26	(2.87, 3.66)	
Any Health insurance					<0.0001
No, has no coverage	9.20	(8.79, 9.62)	5.46	(4.88, 6.03)	
Yes, has coverage	92.79	(90.37, 91.20)	94.54	(93.97, 95.11)	
Has Private Insurance					<0.0001
No	42.70	(41.94, 43.46)	50.52	(49.20, 51.85)	
Yes	57.29	(56.54, 58.06)	49.40	(48.15, 50.99)	
Has Medicaid Insurance					<0.0001
No	87.48	(87.01, 87.95)	79.37	(78.29, 80.44)	
Yes	12.52	(12.05, 12.98)	20.63	(19.56, 21.70)	
Has Medicare Insurance					<0.0001
No	57.04	(56.37, 57.73)	42.93	(41.70, 44.17)	
Yes	42.95	(42.27, 43.63)	57.06	(55.82, 58.29)	
Usual Source of Care					<0.0001
There is no place or No	4.47	(4.19, 4.75)	2.46	(2.07, 2.85)	
Yes, has a usual place or Yes	95.53	(95.25, 95.80)	97.54	(97.15, 97.93)	
BMI					<0.0001
Underweight/normal	15.58	(15.14, 16.01)	18.29	(17.33, 19.25)	
Overweight	32.03	(31.43, 32.63)	28.60	(27.47, 29.74)	
Obese	52.38	(51.74, 53.01)	53.09	(51.76, 54.43)	
Health Status					<0.0001
Excellent	6.12	(5.80, 6.43)	2.54	(2.13, 2.94)	
Very Good	19.86	(19.34, 20.39)	9.47	(8.73, 10.21)	
Good	38.78	(38.16, 39.40)	29.01	(27.82, 30.19)	
Fair	26.11	(25.54, 26.68)	34.81	(33.57, 36.04)	
Poor	9.10	(8.72, 9.48)	24.16	(23.00, 25.31)	
Smoking Status					<0.0001
Current smoker	15.79	(15.29, 16.28)	16.67	(15.73, 17.60)	
Former smoker	32.30	(31.67, 32.93)	38.95	(37.70, 40.20)	
Never smoked	51.90	(51.23, 52.57)	44.37	(43.07, 45.67)	
Taking Insulin for Diabetes					<0.0001
No	74.01	(73.46, 74.56)	60.16	(58.88, 61.44)	
Yes	25.98	(25.43, 26.53)	39.83	(38.55, 41.11)	
Functional Limitation due to Diabetes					<0.0001
Not mentioned	85.87	(85.39, 86.34)	74.61	(73.47, 75.75)	
Mentioned	14.13	(13.66, 14.61)	25.39	(24.25, 26.53)	

Table 2 shows the effects of moderate and severe psychological distress as the main predictor for the study. Both those with moderate psychological distress, odds ratio (OR) = 1.31 (95% CI 1.20-1.43) and serious psychological distress, OR = 1.42 (95% CI 1.28-1.58) were more likely to be hospitalized. Other factors strongly associated with hospitalization include taking insulin for diabetes OR = 1.59 (95% CI: 1.49, 1.69), Medicare coverage OR = 1.38 (95% CI: 1.25, 1.53), Medicaid coverage OR = 1.24 (95% CI: 1.11-1.37), bachelor’s degree OR = 1.15 (95% CI 1.05-1.28), master’s degree or higher OR = 1.18 (95% CI 1.04-1.33), and having some college OR = 1.15 (95% CI 1.06-1.24). Being a former smoker OR = 1.29 (95% CI: 1.21, 1.38), factors associated with a lower likelihood of hospitalization included Asian race OR = 0.73 (95% CI: 0.93- 1.07), and any health status other than ‘poor’, such as very good health OR = 1.10 (95% CI: 0.92, 1.34). 

**Table 2:** Binary logistic regression for hospitalization in prior year for characteristics of adults (18+) reporting diabetes mellitus in the National Health Interview Survey, 2000-2018 (n=48,807) P <0.05 statistically significant Northeast: ME, NY, NJ, VT, MA, RI, CT, NW, and PA. North Central/Midwest: IL, IN, IA, KS, MI, MN, MO, NE, ND, OH, SD, and WI. South: DE, FL, GA, MD, NC, SC, VA, WV, AL, KY, MI, TN, AR, LA, OK, and TX. West: AK, AZ, CA, HI, ID, MT, NV, NM, OR, UT, WA, and WY. GED: General Education Diploma

	Odds Ratio	95% Confidence Interval	P-value*
Mental Health Status			<0.0001
None to low psychological distress	Ref.		
Moderate psychological distress	1.31	(1.20, 1.43)	
Serious psychological distress	1.42	(1.28, 1.58)	
Region			0.27
Northeast	1.11	(1.01, 1.22)	
North Central/Midwest	1.13	(1.04, 1.22)	
South	Ref.		
West	0.87	(0.80, 0.94)	
Sex			0.385
Male	0.97	(0.91, 1.04)	
Female	Ref.		
Year			<0.0001
2000	Ref.		
2001	0.92	(0.77, 1.10)	
2002	0.85	(0.71, 1.02)	
2003	0.95	(0.79, 1.14)	
2004	0.81	(0.68, 0.97)	
2005	0.81	(0.68, 0.95)	
2006	0.80	(0.66, 0.96)	
2007	0.78	(0.64, 0.93)	
2008	0.73	(0.60, 0.88)	
2009	0.86	(0.70, 1.01)	
2010	0.78	(0.65, 0.92)	
2011	0.82	(0.69, 0.97)	
2012	0.76	(0.69, 0.97)	
2013	0.72	(0.64, 0.89)	
2014	0.62	(0.60, 0.86)	
2015	0.75	(0.63, 0.89)	
2016	0.68	(0.57, 0.81)	
2017	0.71	(0.60, 0.85)	
2018	0.71	(0.59, 0.84)	
Age			<0.0001
18-34	Ref.		
35-44	0.89	(0.66, 0.94)	
45-54	0.89	(0.68, 0.93)	
55-64	0.77	(0.66, 0.89)	
65-74	0.79	(0.66, 0.95)	
75 and older	1.00	(0.84, 1.20)	
Race			0.0019
White	Ref.		
Black/African American	0.99	(0.93, 1.07)	
Asian	0.73	(0.93, 1.07)	
Other race	0.95	(0.81, 1.11)	
Hispanic			<0.0001
No, not of Hispanic ethnicity	Ref.		
Yes, of Hispanic ethnicity	0.77	(0.70, 0.84)	
Marital Status			0.0324
Married	Ref.		
Separated	1.04	(0.89, 1.23)	
Divorced	1.14	(1.05, 1.24)	
Widowed	1.09	(1.00, 1.18)	
Living with partner	1.05	(0.89, 1.23)	
Never married	0.99	(0.89, 1.10)	
Education Status			<0.0001
Grade 9-12, no diploma or GED	0.94	(0.86, 1.01)	
High school graduate	Ref.		
Some college, no 4-year degree	1.15	(1.06, 1.24)	
Bachelor's degree	1.15	(1.05, 1.28)	
Master's, Professional, or Doctoral Degree	1.18	(1.04, 1.33)	
Federal Poverty Level (FPL)			0.3701
At or above poverty threshold	Ref.		
Below poverty threshold	1.04	(0.95, 1.14)	
Family’s Home			<0.0001
Owned or being bought	Ref.		
Rented	1.20	(1.13, 1.29)	
Other arrangement	1.34	(1.14, 1.57)	
Any Health Insurance			0.0003
No, has no coverage	0.77	(0.67, 0.89)	
Yes, has coverage	Ref.		
Has Private Insurance			<0.0001
No	Ref.		
Yes	1.01	(0.94, 1.09)	
Has Medicaid Insurance			0.6502
No	Ref.		
Yes	1.24	(1.11, 1.37)	
Has Medicare Insurance			<0.0001
No	Ref.		
Yes	1.38	(1.25, 1.53)	
Usual Source of Care			0.0002
There is no place or No	0.70	(0.58, 0.84)	
Yes, has a usual place or Yes	Ref.		
BMI			<0.0001
Underweight/Normal (< 25 kg/m^2^)	Ref.		
Overweight (25.0 – 29.9 kg/m^2^)	0.79	(0.73, 0.86)	
Obese (≥ 30 kg/m^2^)	0.79	(0.73, 0.85)	
Health Status			<0.0001
Excellent	Ref.		
Very Good	1.10	(0.92, 1.34)	
Good	1.67	(1.39, 1.99)	
Fair	2.70	(2.26, 3.23)	
Poor	4.69	(3.88, 5.67)	
Smoking Status			<0.0001
Current smoker	1.01	(0.93, 1.10)	
Former smoker	1.29	(1.21, 1.38)	
Never smoked	Ref.		
Taking Insulin for Diabetes			<0.0001
No	Ref.		
Yes	1.59	(1.49, 1.69)	
Functional Limitation due to Diabetes			<0.0001
Not mentioned	Ref.		
Mentioned	0.20	(0.03, 5.53)	

## Discussion

This analysis demonstrates that those diagnosed with diabetes and living with moderate and serious sociological distress are more likely to have an increased likelihood of hospitalization compared to those with diabetes but with no to low psychological distress. Those with greater educational attainment were more likely to be hospitalized. Individuals who fall under lower household income brackets tend to have more hospitalizations than those who have more resources. Moreover, T2DM, which affects 8% of the population in the United States, is a condition for whom disease management is especially challenging, owing in part to a lack of detection and treatment of co-morbid mental disorders. 

Similarly, this research supports that those on public insurance plans have more hospitalization than those who are not on public insurance plans. Lastly, those who have poor health status or poor health behaviors are shown to have more hospitalizations. There are some differences between this research and the literature, for example, education status. A study reported that those who have low educational status are more likely to have hospitalizations, but our findings differ [20]. likely explanations are that our study included a larger number of covariates and a national sample, compared to California only [21]. Prevention of hospitalization via management of disease is extremely critical to those who suffer from diabetes mellitus or mental illness alone, but even more so for individuals who suffer from both comorbidities.

T1DM is often unpreventable. On the other hand, T2DM is often preventable. Risk factors of T2DM include excess weight gain, inactivity, and high blood pressure [22]. Family history, neurochemical or hormonal imbalance, stressors of life, life-altering circumstances, traumatic experiences, and chronic medical conditions are all potential risk factors of mental illness [23]. Since there have been rising rates of diabetes mellitus diagnoses, there have been programs and policies in place to reduce obesity, most notably through the CDC Diabetes Control Program [24]. Unfortunately, the same has not been in place for mental illness. Mental illness is often undiagnosed due to the lack of reporting as a result of cultural and societal stigma, a fact that is especially true for Black Americans [25]. Racial minorities are more often diagnosed with comorbidities, specifically with obesity-related diseases such as diabetes options [26]. Furthermore, minorities are more often diagnosed with mental illness compared to Whites [27]. 

Minorities and individuals with low socio-economic status are two times more likely to be diagnosed and hospitalized for diabetes and mental illness than non-minorities or affluent individuals. This is partially due to a lack of health education and lack of prevention resources such as green space and healthy food those who suffer from mental illness and diabetes mellitus include: individuals aged 65 or more, low socioeconomic status, and having Black/African American descent. Poor socioeconomic status is defined based on educational attainment and household and individual yearly income [28]. This study found that poorer individuals, individuals of Black/African American descent, poor health status, and poor health behaviors have more hospitalizations than those who are more affluent or White. Others have shown that those with both diabetes mellitus and mental illness are significantly more likely to be hospitalized for potentially preventable causes. Due to a multitude of barriers to health care that these groups encounter, such as financial resources, transportation, or health education, this is particularly true for people who are older, identify as a minority, and have a poor socioeconomic position [29]. 

Stigma plays a significant role in the underdiagnosis and noncompliance of mental illness management, particularly among minority populations. Although our study sheds light on the relationship between mental health diagnoses and coronavirus disease 2019 (COVID-19) outcomes, we recognize that it does not establish causality. Additionally, our research did not explore other potential factors, such as psychological distress and outpatient care, which might also influence COVID-19 outcomes. Previous research has demonstrated the preventive impact of education on healthy coping mechanisms like exercise and therapy. Educating people about mental illness has been shown to reduce judgment and stigma, fostering a supportive culture for individuals to disclose their mental health symptoms [30]. Integrating mental health education and a supportive environment within media, community organizations, workplaces, and universities. A comprehensive societal shift in attitudes toward mental health may be necessary to reduce stigma [31].

Eliminating barriers to healthcare access is a critical concern for health policy in the U.S. Since the implementation of the Patient Protection and Affordable Care Act (ACA) on January 1, 2014, over 20 million residents have gained access to healthcare [32]. However, a discrepancy persists in the perception of healthcare resources between providers and consumers. Studies have indicated inadequate education among patients using hospital systems and the continuum of care [33]. Over 45% of individuals utilizing healthcare systems struggle to navigate the continuum of care, with this percentage increasing when dealing with insurance or healthcare-associated costs [34]. Furthermore, over 55% of minorities, elderly individuals, and those with low socioeconomic status face difficulties in navigating the continuum of care due to factors such as lack of a permanent home or access to resources like a telephone or car [34]. If patients had a better understanding of the roles of inpatient and outpatient therapies and how to navigate the confusing environment in regard to health insurance, resources, and patient-directed management, we might see a reduction in not only the overwhelming burden of hospitalizations, but also in the prevalence of conditions which prompt these visits. Potential solutions for this include health education and programs and coordinators to ensure continuum of care for those who have complex conditions and comorbidities such as diabetes mellitus and mental illness. Our research highlights the crucial role of healthcare access in determining hospitalization likelihood. Individuals with higher incomes, who may have private health insurance, are less likely to be hospitalized compared to those with Medicare or Medicaid insurance. Although health literacy is essential, our findings indicate no significant difference in education distribution between hospitalized and non-hospitalized individuals. In fact, higher education levels may even correlate with a higher risk of hospitalization due to socioeconomic factors like income and healthcare access.

Health illiteracy is one of the biggest barriers to prevention of disease and compliance with disease management [35]. The results of our study showed a weak association between education level and the risk of hospitalization. This finding may seem surprising given the importance of health literacy in promoting good health outcomes. It is possible that factors other than formal education, such as individual health behaviors and social determinants of health, have a greater influence on hospitalization risk. Nevertheless, further research is needed to explore the complex relationship between education, health literacy, and hospitalization [36]. As indicated, many of those diagnosed with diabetes mellitus are minorities, elderly, and have poor socioeconomic status. To reduce this inequality, health leaders, practitioners, and educators must educate on prevention and management of mental illness and diabetes mellitus in an understandable way. It was believed that the average adult literacy is of a third grader, but new research indicates it may be as low as a first grader [37]. It is critical for material to be presented to patients suffering from mental illness and diabetes in a way that they understand proper treatment maintenance in order to prevent future hospitalization. 

Compliance with treatment maintenance may depend on the relationship between provider and patient. Since diagnoses of mental illness and diabetes mellitus occur more frequently amongst minorities and individuals with low socioeconomic status, it is particularly important that providers have good relationships with such individuals. Unfortunately, biases, prejudices, and stereotypes held by providers often result in lower quality of care [38]. One factor may be that medical training often does address cultural competencies [38]. In order to reduce this inequity in care, healthcare providers should receive greater cultural competency training. This should lead to better treatment compliance and maintenance, especially amongst minorities who suffer from comorbidities such as diabetes mellitus and mental illness, therefore preventing hospitalization due to complications. 

Strengths and limitations

Limitations include that NHIS is self-reported, thus participants may have responded inaccurately or chosen not to respond. Self-reported surveys can be inaccurate due to the over-response of positive health results and a response of negative health status measures. Another limitation is that we could not differentiate between T1DM and T2DM or among specific mental illness diagnoses. This limits potential programs and policies in order to best serve the population that is affected most. Another limitation is that the reason for hospitalization is unknown, thus participants may have been hospitalized for reasons not related to diabetes or mental illness. Unfortunately, NHIS does not measure the cost of hospitalization in dollars. Strengths of this study include a large sample size, a randomly selected, nationally representative sample, and confidence in the stability of findings as this survey has been conducted for more than 50 years. 

## Conclusions

This study found a significant association between psychological distress and hospitalization among adults with diabetes. Individuals with moderate or severe psychological distress are more likely to be hospitalized. Understanding the factors contributing to this increased hospitalization burden can help healthcare providers and policymakers develop targeted interventions to reduce hospitalizations and healthcare costs.
